# New, rapid method to measure dissolved silver concentration in silver nanoparticle suspensions by aggregation combined with centrifugation

**DOI:** 10.1007/s11051-016-3565-0

**Published:** 2016-08-29

**Authors:** Feng Dong, Eugenia Valsami-Jones, Jan-Ulrich Kreft

**Affiliations:** 1 Institute of Microbiology and Infection, School of Biosciences, University of Birmingham, Edgbaston, Birmingham, B15 2TT UK; 2 School of Geography, Earth and Environmental Sciences, University of Birmingham, Edgbaston, Birmingham, B15 2TT UK

**Keywords:** Silver nanoparticles, Sedimentation, Aggregation, Separation, Centrifugation, Nanotoxicology, Environmental and health effects

## Abstract

It is unclear whether the antimicrobial activities of silver nanoparticles (AgNPs) are exclusively mediated by the release of silver ions (Ag^+^) or, instead, are due to combined nanoparticle and silver ion effects. Therefore, it is essential to quantify dissolved Ag in nanosilver suspensions for investigations of nanoparticle toxicity. We developed a method to measure dissolved Ag in Ag^+^/AgNPs mixtures by combining aggregation of AgNPs with centrifugation. We also describe the reproducible synthesis of stable, uncoated AgNPs. Uncoated AgNPs were quickly aggregated by 2 mM Ca^2+^, forming large clusters that could be sedimented in a low-speed centrifuge. At 20,100g, the sedimentation time of AgNPs was markedly reduced to 30 min due to Ca^2+^-mediated aggregation, confirmed by the measurements of Ag content in supernatants with graphite furnace atomic absorption spectrometry. No AgNPs were detected in the supernatant by UV–Vis absorption spectra after centrifuging the aggregates. Our approach provides a convenient and inexpensive way to separate dissolved Ag from AgNPs, avoiding long ultracentrifugation times or Ag^+^ adsorption to ultrafiltration membranes.

**Electronic supplementary material:**

The online version of this article (doi:10.1007/s11051-016-3565-0) contains supplementary material, which is available to authorized users.

## Introduction

Nanomaterials (NMs) have received increasing attention due to their distinctive physicochemical properties at nanosize (Adeleye et al. 2016; Kim et al. 2010; Lohse and Murphy 2012), especially for their medical application potential, as multi-drug resistant pathogens become ever more frequent (Meredith et al. 2015; Morones-Ramirez et al. 2013; Piddock 2012; Sprenger and Fukuda 2016). Silver nanoparticles (AgNPs) are widely used nanomaterials due to their toxic effects on microorganisms (Chaloupka et al. 2010; Eckhardt et al. 2013; Morones-Ramirez et al. 2013). However, the mechanisms involved in their toxicity to microorganisms are still unclear. The antibacterial activitie**s** of AgNPs are generally thought to be indirectly mediated by the release of silver ions (Ag^+^) (Leclerc and Wilkinson 2014; Shen et al. 2015; Xiu et al. 2012), but some studies have suggested nanoparticles themselves can play a direct role in toxicity to bacteria (Ivask et al. 2013; McQuillan and Shaw 2014) because of their potential to directly interact with microbial components (Eckhardt et al. 2013) and their large proportion of reactive surface sites compared to bulk materials (Oberdörster et al. 2005). It is therefore essential to differentiate ionic and nano-Ag and measure the concentrations of different Ag species to understand their bactericidal effects.

Silver nanoparticles become oxidized, releasing Ag^+^ in aquatic environments, when oxygen is available (Liu and Hurt 2010; Peretyazhko et al. 2014; Zhang et al. 2011). The dissolved Ag usually coexists with nanoparticles during storage (Loza et al. 2014). Quantifying Ag species in AgNP dispersions, however, is quite challenging due to the difficulty of separating dissolved Ag^+^ from AgNPs. Ultracentrifugation and ultrafiltration are the methods used routinely. Both have their limits. In ultracentrifugation, precipitating the tiny nanoparticles requires large centrifugal forces and long running times (Mostowfi et al. 2009), which might be problematic when AgNPs continuously release Ag^+^ in an oxic environment. Sorption of Ag^+^ to membranes may occur when ultrafiltration (Kennedy et al. 2010; Leclerc and Wilkinson 2014) or dialysis is used. Ion-selective electrodes (ISE) can be used to measure free Ag^+^ concentration in bulk liquid but not the total dissolved Ag^+^ (Shen et al. 2015). Emerging techniques such as single particle inductively coupled plasma mass spectrometry (SP-ICP-MS) and asymmetrical flow field-flow fractionation coupled with inductively coupled plasma mass spectrometry (AF4-ICP-MS) are complex to operate and standardize (Mitrano et al. 2012; Pace et al. 2012).

In this study, we developed a convenient and reliable method to quantify dissolved Ag in Ag^+^/AgNP mixtures by combining aggregation with centrifugation. The aggregation of AgNPs in different concentrations of Ca(NO_3_)_2_ solutions was investigated to examine how the aggregation mediated by Ca^2+^ facilitated the sedimentation of AgNPs during centrifugation. By comparing with ultrafiltration, we demonstrated that this new method provided a simple, fast and effective way to monitor dissolved Ag in AgNP suspensions.

## Materials and methods

### Synthesis of uncoated AgNPs

Uncoated AgNPs were produced by the solution-phase method (Polte et al. 2012; Van Hyning and Zukoski 1998). Silver nitrate (AgNO_3_, Sigma-Aldrich) was used as the precursor and reduced by sodium borohydride (NaBH_4_, Sigma-Aldrich) to form AgNPs at room temperature (19 ± 4 °C). All the glassware for AgNPs synthesis was soaked in 10 % HNO_3_ overnight and rinsed with copious amounts of deionized (DI) water (18.2 mΩ, Millipore), followed by drying in an ambient environment. The AgNO_3_ solution (100 mL, 0.12 mM) was poured into the NaBH_4_ solution (100 mL, 3 mM) in a 500-mL beaker. The NaBH_4_ concentration was in 25-fold excess. The mixture was homogenized by magnetic stirring (1200 rpm). The NaBH_4_ solution was freshly prepared to reduce the degradation resulting from its reaction with water to produce H_2_ and BO_4_^−^. The solution turned to grey within a few seconds after mixing and changed to light yellow after a few minutes. As the reaction continued, the colour slowly changed to dark yellow at ~25 min and back to yellow. After 1 h, the stirring was stopped, and the solution was stored for 24 h in the dark at room temperature. Finally, the stirring bar was removed and the AgNP suspension was transferred into glass bottles (250 mL, Duran) and stored at 4 °C in the dark.

### Characterization of uncoated AgNPs

The localized surface plasmon resonance (LSPR) of uncoated AgNP suspensions was measured by UV–Vis spectrometry (UV–Vis 6800, Jenway, Staffordshire, UK). The relationship between LSPR signal and AgNP concentration was obtained by measuring the absorption spectra of a dilution series of AgNP suspensions.

The size distribution of AgNPs was measured by dynamic light scattering (DLS) (Zetasizer Nano, Malvern Instruments, Malvern, UK) and differential centrifugal sedimentation (DCS) (DC24000, CPS Instruments Europe, Oosterhout, Netherlands).

The dissolved Ag fraction in AgNP suspensions was obtained by filtering the suspensions through a 3-kDa membrane filter (Amicon Ultra-15 Centrifugal Filter Unit, Millipore (UK) Limited, Hertfordshire, UK) at a centrifugal force of 4000*g* for 20 min at 4 °C. The filtrate was collected and stored at 4 °C for future analysis. Total Ag concentration was measured by acidifying 1 mL AgNP suspension with 9 mL 70 % HNO_3_ (w/w) overnight at room temperature. The digested suspension was diluted with DI H_2_O (18.2 mΩ, Millipore) to a final HNO_3_ concentration of 0.2 % (w/v). The silver content was measured by graphite furnace atomic absorption spectrometry (GFAAS) (AAnalyst 600, PerkinElmer Instruments, Massachusetts, USA). A silver concentration series (0–25 μg/L) was obtained by diluting a standard AgNO_3_ solution (1000 μg/mL Ag, PerkinElmer Life and Analytical Sciences, Shelton, USA) with 0.2 % HNO_3_ concentration (w/v). Those standards were measured together with samples to obtain a calibration curve for calculating sample concentrations.

The morphology of AgNPs was imaged by transmission electron microscopy (TEM, JEOL 1200EX, Tokyo, Japan). About 20 μL AgNP suspension was loaded onto TEM grids (CF300-Cu Grids, Electron Microscopy Sciences, Pennsylvania, USA), followed by drying at room temperature. In order to reduce aggregation of AgNPs after loading the grids, grids were coated with 20 μL 100 mg/L polylysine, which carries many positive charges while the AgNPs have a negative surface charge. After 1 h, the grids were rinsed with DI water followed by drying. The morphology of AgNPs in Ca(NO_3_)_2_ solution was analysed by adding 100 mg/L bovine serum albumin (BSA) to stabilize the aggregates before loading the sample on a TEM grid without polylysine. BSA can be used to preserve the nanoparticle state in electrolyte solutions to avoid artefacts of drying (Michen et al. 2015).

### Determining aggregation kinetics of AgNPs in Ca(NO_3_)_2_ solutions

The long-term aggregation of AgNPs in Ca(NO_3_)_2_ solution was followed for 96 h after mixing 20 mL of a AgNP suspension with the same volume of a 2 mM Ca(NO_3_)_2_ solution by vortexing in screw-cap glass vials (Bijou, capacity 46 mL) and allowing the mixture to settle on a laboratory bench at room temperature (17 ± 1 °C). The liquid from the top layer (5 mL) was taken for recording UV–Vis absorption spectra after 0, 1, 6, 24, 48 and 96 h.

The short-term aggregation kinetics were monitored for 0.5–2 h after mixing AgNP and Ca(NO_3_)_2_ solutions by recording the hydrodynamic size of aggregates by DLS in real time. The AgNP suspension (0.5 mL, total Ag 5012 ± 75 μg/L, dissolved Ag 28 ± 0.5 μg/L) was mixed with 0.5 mL of different concentrations of Ca(NO_3_)_2_ in a disposable plastic cuvette and immediately placed in the Zetasizer Nano to record the Z-average diameter. Temperature was controlled at 25 °C. Different concentrations of AgNPs that were obtained by diluting the AgNP stock by DI H_2_O were mixed with 0.5 mL of 2 or 20 mM Ca(NO_3_)_2_ in the same way to investigate the aggregation of dilute AgNP suspensions.

### Centrifugation of AgNPs in Ca(NO_3_)_2_

The sedimentation speeds of AgNPs with or without Ca^2+^-mediated aggregation were compared at the same centrifugal force. AgNPs were aggregated by mixing 10 mL AgNP suspension with 10 mL Ca(NO_3_)_2_ solution (2 mM). After 10 min, the AgNPs/Ca(NO_3_)_2_ mixture was aliquoted into several centrifuge tubes (1 mL for each tube). Diluting 10 mL of the same AgNP suspension in 10 mL DI H_2_O was used as control. Centrifugation was undertaken to sediment aggregates or individual AgNPs at a centrifugal force of 20,100*g* (Centrifuge 5417 C, Eppendorf, Engelsdorf, Germany), leaving dissolved Ag in the supernatant. To investigate the extent to which pre-aggregation can reduce centrifugation time, one tube from each treatment was taken to measure the Ag content in the supernatant (0.4–0.5 mL) at 0, 0.5, 1, 1.5, 2, 2.5, 3 and 4 h. The supernatants were acidified to 1 % HNO_3_ (w/v) overnight (more than 12 h) at 80 °C, and further diluted to a final HNO_3_ concentration of 0.2 % (w/v) for Ag concentration measurement by GFAAS.

### Measurement of AgNP content in supernatant after centrifugation of AgNPs in Ca(NO_3_)_2_

Suspensions of AgNPs (0.5 mL) were mixed with Ca(NO_3_)_2_ (0.5 mL) in 1.5-mL microcentrifuge tubes (safe-lock microcentrifuge tubes, Eppendorf, Germany) to form large aggregates. After reacting for 10 min, aggregates were centrifuged at 20,100*g* for 30 min. Ten aliquots were processed in parallel. The amount of AgNPs in the supernatant was measured by UV–Vis absorption spectroscopy. The supernatants (0.5 mL) from those ten aliquots were pooled into one tube since 4 mL was the minimum volume required for a UV–Vis absorption measurement with a 10-cm path length quartz cuvette. Although the UV–Vis absorption of nanoparticles depends on their size, shape, surface coating, aggregation states and surrounding environmental conditions, it can still be used for concentration measurement as long as the particles and surrounding environmental conditions are similar (Hendel et al. 2014). A low concentration of AgNPs (<10 μg/L) can be detected by UV–Vis spectrometry using a 10-cm path length quartz cuvette. The peak absorbance, typically between 390 and 400 nm, was used to quantify the AgNPs, and the absorbance at longer wavelengths (500–700 nm) was used to monitor the aggregates (Gorham et al. 2014).

### Measuring dissolved Ag in AgNPs suspension with aggregation–centrifugation or ultrafiltration

Different concentrations of AgNPs were prepared by diluting the AgNP stock with DI H_2_O. The total Ag concentration of the AgNP stock was 5012 ± 75 μg/L, and it contained around 10 % dissolved Ag. Those diluted AgNP suspensions were aggregated in 2 mM Ca(NO_3_) for 10 min and then centrifuged (20,100*g*, 30 min). The supernatant (0.4–0.5 mL) was collected carefully and stored at −20 °C for dissolved Ag analysis. Each concentration was assayed in duplicate. Ultrafiltration was carried out by filtering the same AgNP suspensions through 3-kDa membrane filters (Amicon Ultra-15 Centrifugal Filter Unit, Millipore (UK) Limited, Hertfordshire, UK). The filtrates were stored at −20 °C. The dissolved Ag concentrations were always analysed by GFAAS.

## Results

### Synthesis of uncoated AgNPs

**Fig. 1:**
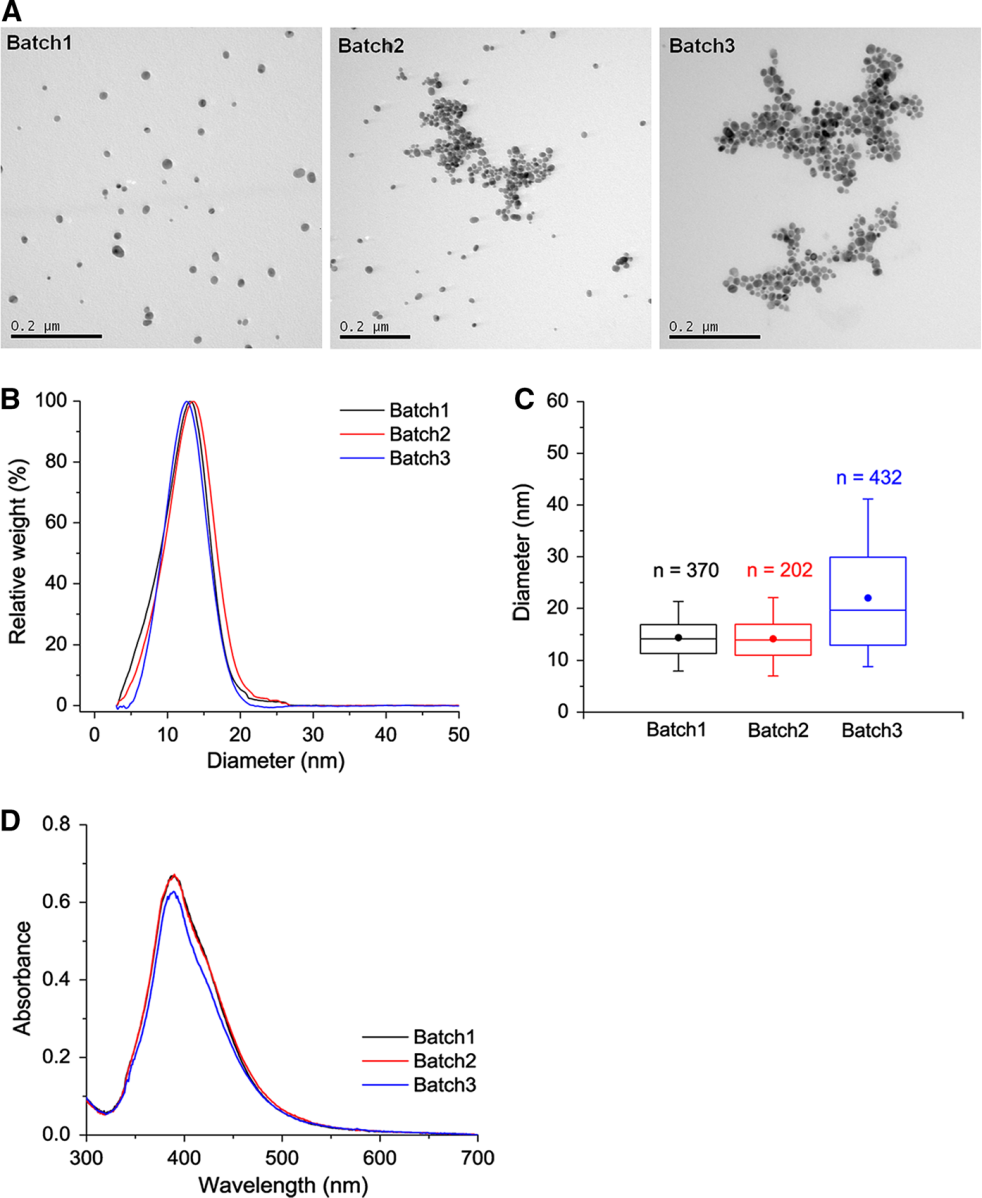
Characterization of uncoated AgNPs and reproducibility of synthesis. **a** Morphology by TEM. **b** Comparison of size distributions measured by DCS. **c** Size distributions analysed by ImageJ (Schneider et al. 2012) based on the TEM images. The *boxes* represent interquartiles, and the *whiskers* represent 5 and 95 percentiles. The points in the *box* are the means, and the *bars* represent the median. **d** UV–Vis absorption spectra 1 day after synthesis

Three batches of AgNP suspensions were synthesized by the same procedure and characterized (Table 1; Fig. 1). They were reasonably monodispersed in H_2_O and had a spherical shape (Fig. 1a). The size distributions of the three batches were similar. Measured by DCS, they showed the same peak diameter of 13 ± 1 nm (Fig. 1b). Measured by TEM, the diameters of more than 83 % of the counted AgNPs ranged between 10 and 40 nm (Fig. 1c). The three batches also had similar UV–Vis absorption spectra and the same peak absorbance wavelength (390 ± 1 nm) (Fig. 1d).

**Table 1:** Characteristics of uncoated AgNP suspensions

pH	Diameter (nm)			Zeta potential in DI water (mV)
	TEM	DLS	DCS	
9.6 ± 0.3	17 ± 4.5	27 ± 4.2	13 ± 0.5	−40 ± 17

All data are shown as mean ± standard deviation of three batches produced with the same procedure. Diameters were measured by TEM, DLS (peak size based on intensity distribution) and DCS (peak size based on relative weight distribution)

### AgNPs aggregate in Ca(NO_3_)_2_ solutions

**Fig. 2:**
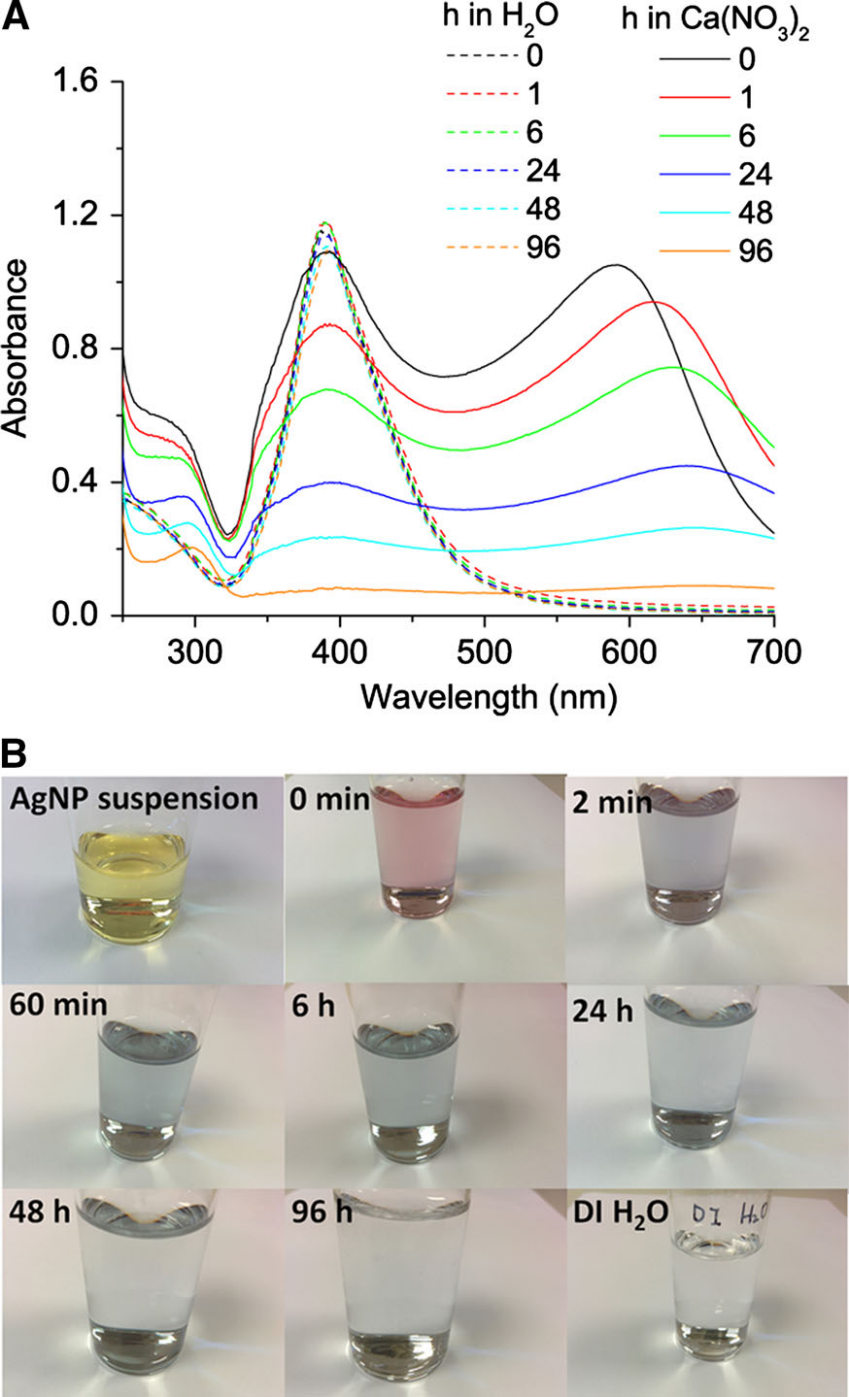
Aggregation of AgNPs in 2 mM Ca(NO_3_)_2_ solution. **a** UV–Vis absorption spectra of AgNP suspensions in H_2_O or 2 mM Ca(NO_3_)_2_ as a function of time, from 0 to 96 h. **b** Photographs of the AgNP dispersion in 2 mM Ca(NO_3_)_2_ taken over time

The small particle size made complete separation of AgNPs from ionic Ag difficult. Therefore, we investigated whether the particles would aggregate quickly in the presence of Ca(NO_3_)_2_ and could thus be separated by precipitation (Fig. 2). Individual AgNPs were stable in H_2_O as the UV–Vis absorption spectra did not change significantly during 96 h, indicating lack of aggregation (Fig. 2a). The absorbance of AgNPs (at the wavelength of maximal absorbance) was proportional to the concentration of AgNPs and can therefore be used to measure the concentration of AgNPs (Fig. S1). The AgNP suspensions aggregated quickly in the Ca(NO_3_)_2_ solutions. The top layers of the mixtures were sampled for UV–Vis absorbance measurements. The red shift of the peak absorbance wavelength from 600 to 650 nm accompanied by the decreasing absorbance at 400 nm suggested that the aggregation took place during the first 6 h. The declining absorbance at both 400 and 650 nm from 6 to 96 h indicated the sedimentation of the aggregates. After 96 h, the absorbance in the range of 350–700 nm declined to <0.09, suggesting that most of the AgNPs and aggregates sedimented. This aggregation and precipitation of AgNPs in Ca(NO)_3_ solution were also confirmed by the colour transformations. The colour of the AgNP suspension changed from yellow to pink in a few seconds (referred to as 0 min) after mixing the AgNP suspension with Ca(NO_3_)_2_ solution, followed by light blue during the first hours (Fig. 2b). Afterwards, the colour strength declined slowly, and the dispersion was as colourless as DI H_2_O at 96 h while a dark precipitate had formed at the bottom.

### Concentration dependence of aggregation of AgNPs in Ca(NO_3_)_2_

**Fig. 3:**
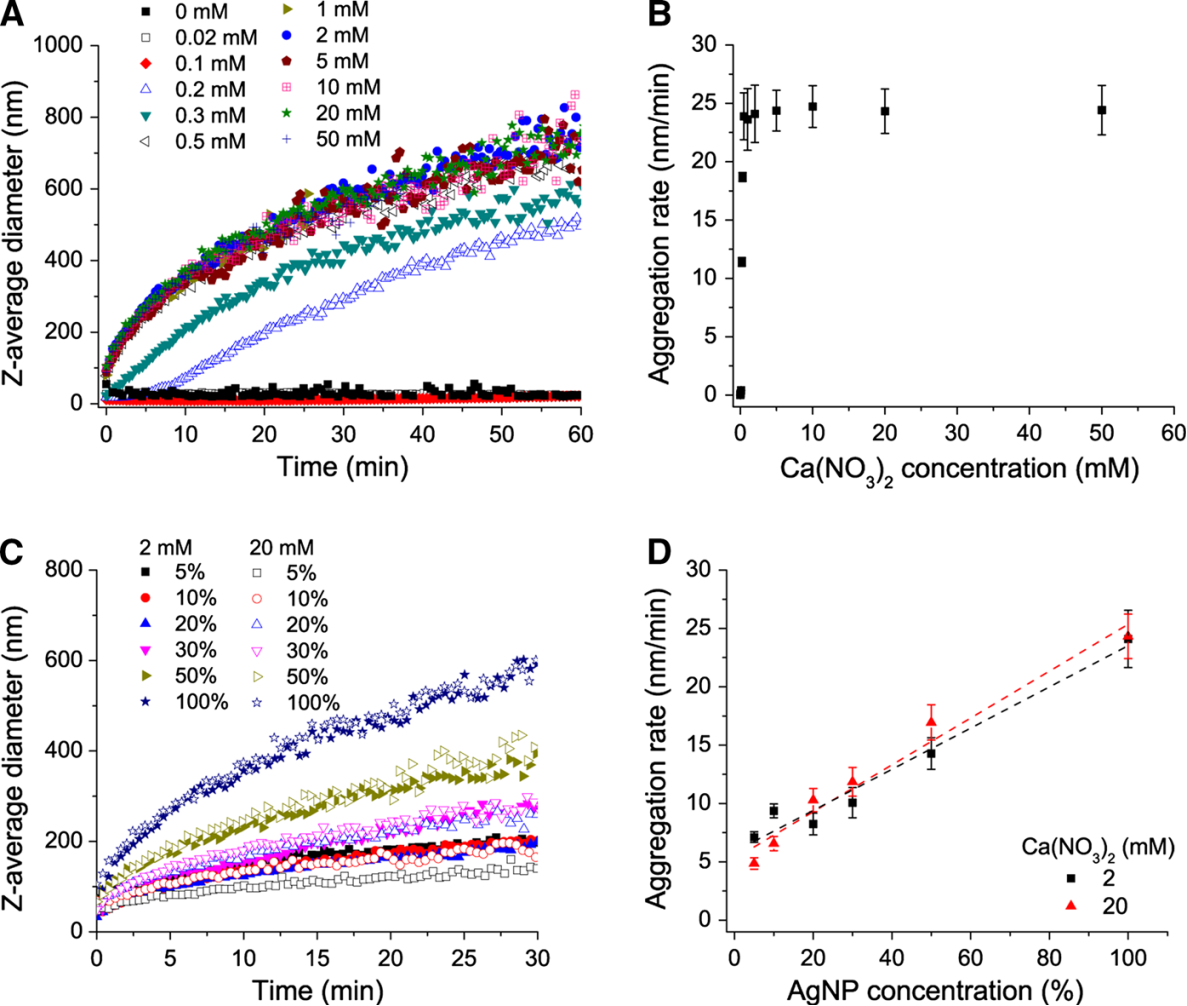
Aggregation kinetics of AgNPs in Ca(NO_3_)_2_ solution. **a** Dependence of AgNP aggregation kinetics on Ca(NO_3_)_2_ concentration. **b** Aggregation rates calculated by linear regression of the first aggregation phase shown in (**a**). *Error bars* indicate 95 % confidence intervals. **c** Aggregation kinetics of different concentrations of AgNPs in 2 and 20 mM Ca(NO_3_)_2_. **d** Aggregation rates calculated by linear regression of the first aggregation phase in 2 and 20 mM Ca(NO_3_)_2_ shown in (**c**). *Error bars* indicate 95 % confidence intervals

**Fig. 4:**
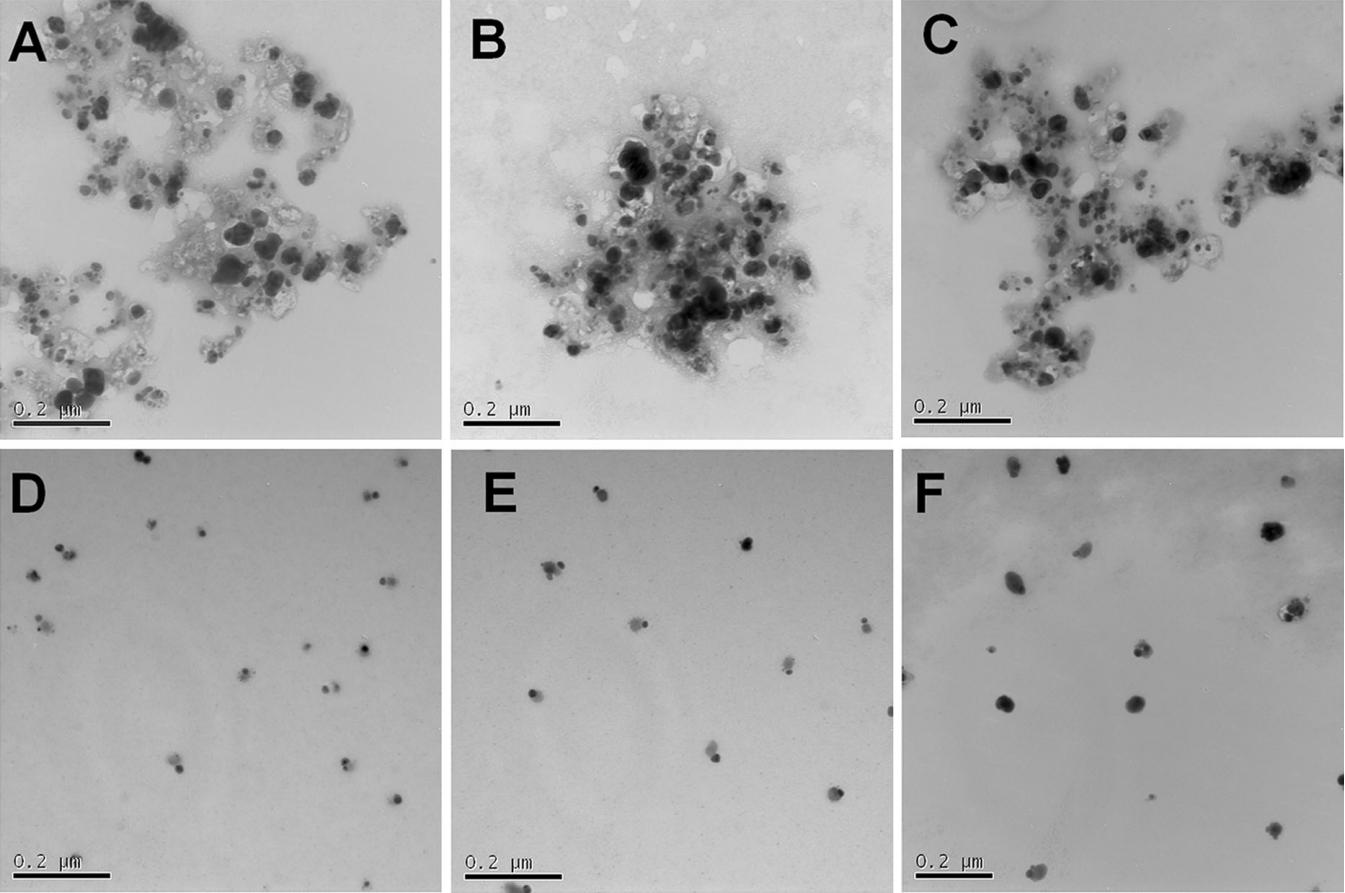
TEM images of AgNPs in 2 mM Ca(NO_3_)_2_ (**a**–**c**) or H_2_O (**d**–**f**)

We investigated the Ca(NO_3_)_2_ concentration dependence of AgNP aggregation. The aggregation process appeared to be divided into two phases, the faster initial aggregation phase presumably corresponds to aggregation of individual AgNPs or small clusters and the second, slower aggregation phase corresponds to aggregation of larger aggregates. The aggregation rate of AgNPs in the initial aggregation phase markedly increased from close to zero to 11 nm/min when the Ca(NO_3_)_2_ concentration increased from 0.1 to 0.2 mM (Fig. 3a). It did not increase further once the Ca(NO_3_)_2_ concentration was higher than 0.5 mM (Fig. 3b). Hence, 2 mM Ca(NO_3_)_2_ ensured sufficient aggregation. Following this, we investigated the AgNP concentration dependence of aggregation in 2 and 20 mM Ca(NO_3_)_2_. Higher AgNP concentrations favoured aggregation, and larger aggregates were formed, both in 2 and 20 mM Ca(NO_3_)_2_ (Fig. 3c). When the AgNP concentration increased from 250 to 5012 μg/L, the aggregation rate increased from 5 to 24 nm/min. Linear regression was carried out to assess the relationship between aggregation rate and AgNP concentration (Fig. 3d). The regression slopes ± SE in nm/min were 17.7 ± 1.6 (*p* value =3.62 × 10^−4^) and 20.1 ± 1.7 (*p* value =3.25 × 10^−4^) in 2 and 20 mM Ca(NO_3_)_2_, respectively. Increasing the Ca(NO_3_)_2_ concentration from 2 to 20 mM increased the aggregation rate only slightly. This suggested that a concentration of 2 mM Ca(NO_3_)_2_ was sufficient to trigger AgNP aggregation, even when the concentration of AgNPs in suspension was quite low. Aggregation of AgNPs in Ca(NO_3_)_2_-containing media led to the formation of large clusters (Fig. 4a–c) compared to single scattered AgNPs in H_2_O (Fig. 4d–f).

### Time required to sediment AgNPs after aggregation

**Fig. 5:**
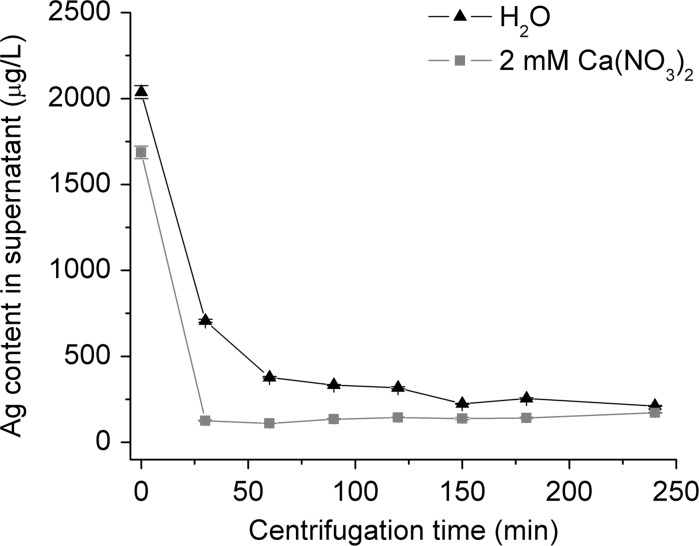
Ag content in supernatants after centrifuging AgNPs in 2 mM Ca(NO_3_)_2_ or H_2_O. *Error bars* represent the standard deviations of three measurements of the sample by GFAAS

The change of Ag content in the top layer of AgNP/Ca(NO_3_)_2_ and AgNP/H_2_O mixtures during centrifugation was measured. After 30 min of centrifugation, 66 % of the AgNPs had sedimented towards the bottom. A slower decrease in Ag content in the supernatant was observed after this steeper initial decline (Fig. 5). At least 4 h was required to sediment all AgNPs in H_2_O. When AgNPs were aggregated by Ca(NO_3_)_2_, all the AgNPs sedimented to the bottom of the vessel in 30 min of centrifugation. Prolonged centrifugation did not further decrease the Ag content in the supernatant (Fig. 5). Hence, 30 min was sufficient to precipitate virtually all AgNPs in 2 mM Ca(NO_3_)_2_ solutions.

### Sedimentation of Ca^2+^-aggregated AgNPs is complete

**Fig. 6:**
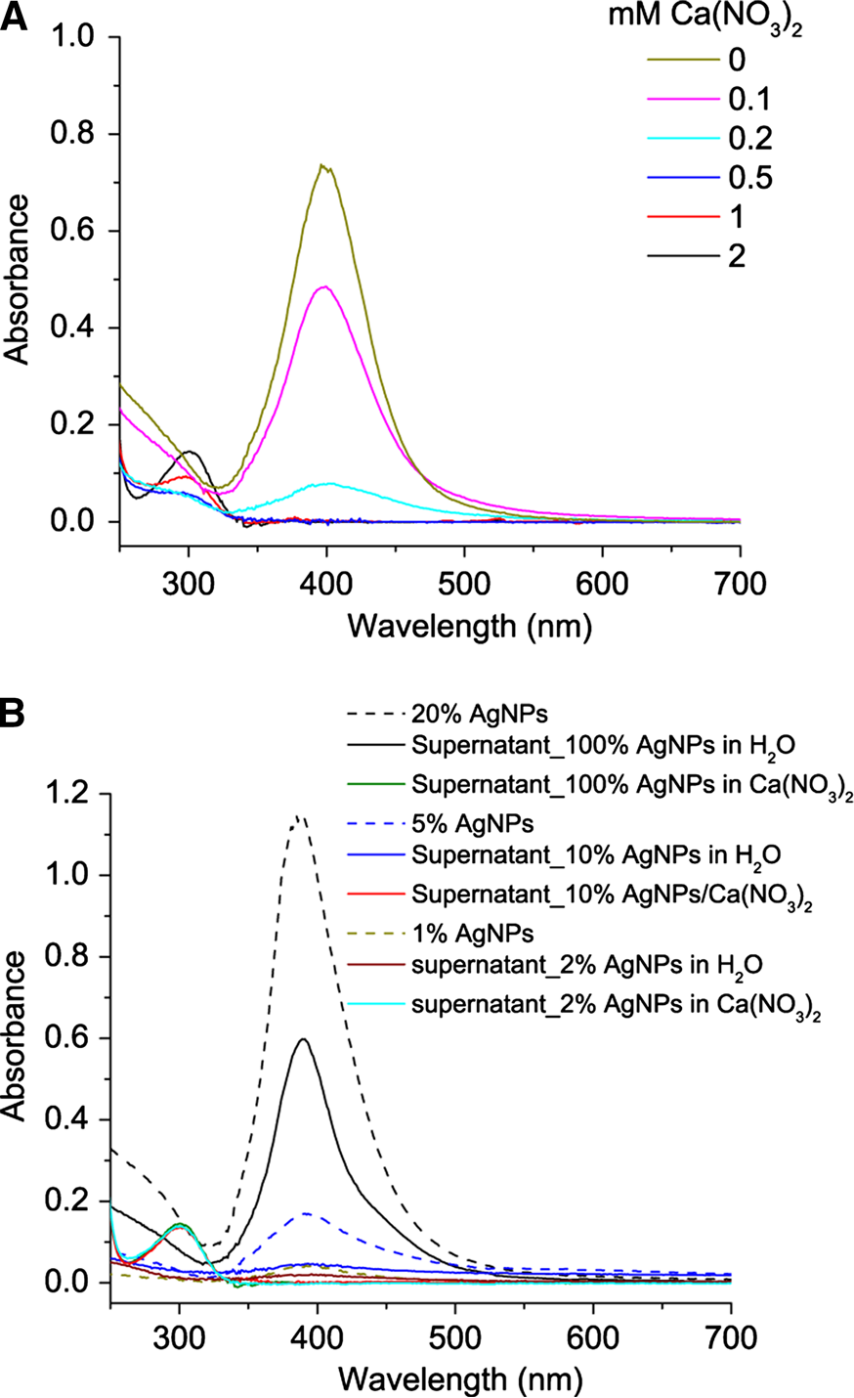
**a** UV–Vis absorption spectra of supernatant after centrifugation of AgNPs in various concentrations of Ca(NO_3_)_2_. **b** The same for various concentrations of AgNPs in 2 mM Ca(NO_3_)_2_ or H_2_O. Since the concentration of the AgNP stock (total Ag 5012 ± 75 μg/L; dissolved Ag 283 ± 13 μg/L) was too high for recording the spectrum, it was diluted to 20 %

To investigate the effects of aggregation on nanoparticle sedimentation, AgNPs were aggregated in different concentrations of Ca(NO_3_)_2_ before centrifugation. The absorption spectra of the supernatants had the same shape as absorption spectra of monodispersed AgNP suspensions, suggesting that the absorption of supernatants was due to individual AgNPs in the supernatant (Fig. 6a). Therefore, absorbance at the wavelength of maximal absorption can be used to quantify AgNPs. Increasing the Ca(NO_3_)_2_ concentrations from 0 to 0.5 mM increased the reduction of the AgNP content in the supernatant from 34 % of initial AgNPs to below detection level. Concentrations of Ca(NO_3_)_2_ larger than 0.5 mM ensured that all AgNPs aggregated, leading to complete sedimentation as shown by negligible absorption in the long wavelength region (550–700 nm) (Fig. 6a).

We investigated the efficiency of combining aggregation with centrifugation to precipitate different concentrations of AgNPs (100, 10 and 2 % of the concentration of the AgNP stock suspensions). Supernatants did not show any absorption from 350 to 700 nm after aggregating those concentrations of AgNPs in 2 mM Ca(NO_3_)_2_. Instead, 21–50 % of AgNPs remained in the supernatant without Ca^2+^ (Fig. 6b). Therefore, 2 mM Ca(NO_3_)_2_ was sufficient to aggregate even the very dilute AgNPs.

### Measuring dissolved Ag in AgNP suspensions by aggregation or ultrafiltration

**Fig. 7:**
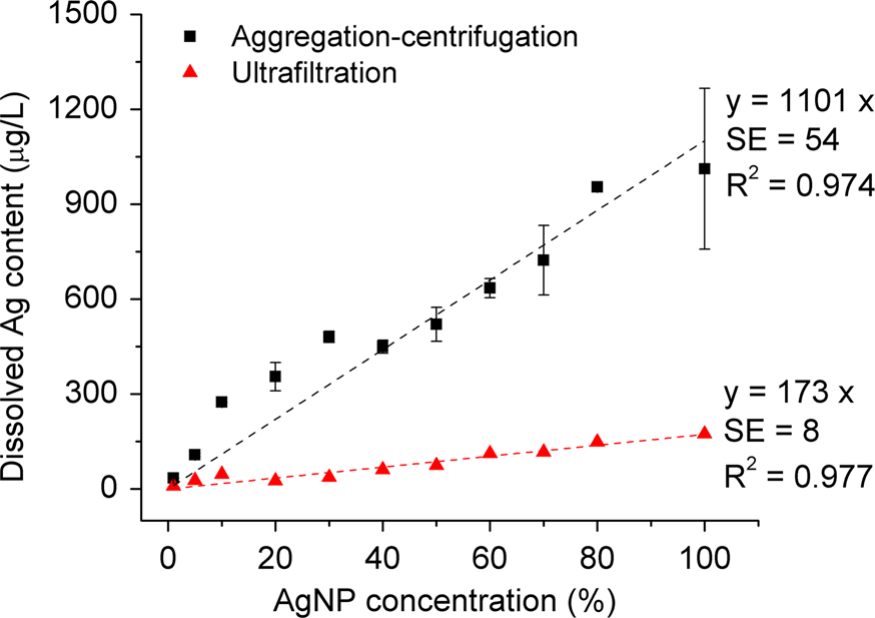
Dissolved Ag contents measured by aggregation–centrifugation or ultrafiltration. *Error bars* indicate standard deviations (*n* = 2). Linear regression of dissolved Ag concentration versus AgNP concentration confirmed the proportionality. SE indicates standard error of the slopes (the intercept was set to 0)

The AgNP samples containing various concentrations of dissolved Ag were obtained by diluting the AgNP stock with H_2_O. The dissolved Ag concentrations in these samples should therefore be proportional to the AgNP concentration. This was confirmed by linear regression as the adjusted R^2^ were high in each case (0.974 for aggregation–centrifugation and 0.977 for ultrafiltration) (Fig. 7). However, the slope of the regression line for aggregation–centrifugation was 1101 ± 54, i.e., 6.4 times larger than the slope for ultrafiltration (173 ± 8). This means that 6.4-fold more dissolved Ag was detected by aggregation–centrifugation. A partial explanation for this difference was the loss of Ag we observed during ultrafiltration (Fig. S2). The amount of Ag lost was proportional to the initial Ag^+^ concentration before filtration. Although Ag recovery gradually improved over five cycles of filtration of the same AgNO_3_ solution, there was still a loss of 39–44 % of Ag in the last cycle (Fig. S2).

## Discussion

### Formation of stable, uncapped AgNPs

Our study demonstrated the successful synthesis of monodispersed uncoated AgNPs (Fig. 1; Table 1). The formation of uncoated AgNPs is controlled by the aggregative growth of small AgNPs (Polte et al. 2012; Van Hyning et al. 2001), but aggregation of the NPs can lead to the failure of synthesis (Mulfinger et al. 2007). Frequently, the electrolyte with highest ionic strength contributes most to the aggregation (Huynh and Chen 2011; Li et al. 2012). As expected, by decreasing initial AgNO_3_ and NaBH_4_ concentrations to reduce the ionic strength and increasing the NaBH_4_/AgNO_3_ concentration ratio so more BH_4_^−^ is available to stabilize uncoated AgNPs (Van Hyning and Zukoski 1998), we managed to produce stable uncoated AgNPs. The process was reproducible, and the three batches of AgNPs did not differ significantly in their aggregation in Ca(NO_3_)_2_ or sedimentation by centrifugation.

### Aggregation of AgNPs in Ca(NO_3_)_2_ solution

According to classic colloidal theory, interactions between nanoparticles are determined by van der Waals forces, electrostatic repulsive forces and steric effects due to surface stabilizers and solvation effects (Nel et al. 2009). For uncoated AgNPs, negatively charged ions such as BH_4_^−^ adsorb on the nanoparticles with counter ions enriched near the surface (Mulfinger et al. 2007; Pfeiffer et al. 2014). Positively charged Ca^2+^ ions screen the negative surface charge of AgNPs, thus decreasing surface energy. Aggregation of AgNPs occurs when the kinetic energy of Brownian motion overcomes the nanoparticle–nanoparticle energy barrier, which is reduced in Ca(NO_3_)_2_ solution (Zhang et al. 2012). The structure of aggregates formed, such as configuration and monomer numbers, depends on nanoparticle concentration, size distribution, surface coating and conditions in the surrounding liquid (Baalousha et al. 2013; El Badawy et al. 2010). The gap between clustered nanoparticles can be as narrow as a few nanometres (Silvera Batista et al. 2015). Under normal gravitational force, small metallic nanoparticles take days or weeks to settle a few millimetres (Alexander et al. 2013). In contrast, the AgNPs completely sedimented to the bottom in 2 mM Ca(NO_3_)_2_ solution within 4 days (Fig. 2), suggesting that aggregation facilitates sedimentation of AgNPs.

The aggregation rate of AgNPs increased markedly when the Ca(NO_3_)_2_ concentration was increased from 0.1 to 0.2 mM, reaching a maximum rate at 0.5 mM (Fig. 3a, b). This suggests that ~0.2 mM was the concentration separating the slow and fast aggregation regimes, which is known as the critical coagulation concentration (CCC). The aggregation rate was proportional to AgNP concentration in both 2 and 20 mM Ca(NO_3_)_2_ solutions (Fig. 3c, d). Increasing AgNP concentration will increase the number of AgNPs with high enough kinetic energy to overcome the energy barrier upon collision and thereby increase the aggregation rate. Notably, 2 and 20 mM Ca(NO_3_)_2_ aggregated equivalent concentrations of AgNPs equally fast (Fig. 3d), suggesting that any concentration above the CCC is sufficient to ensure optimum aggregation of AgNPs.

### Efficiency of AgNP sedimentation in Ca(NO_3_)_2_ solution by centrifugation

It is predicable that centrifugation will shorten the sedimentation time of AgNP aggregates. As the sedimentation velocity is proportional to the square of nanoparticle diameter, the sedimentation distance declines dramatically with decreasing size (Bonaccorso et al. 2013). Consequently, it takes exceedingly long times to sediment small AgNPs completely (Fig. S3). Therefore, centrifugation of monodispersed AgNPs in H_2_O will especially increase the sedimentation of the larger AgNPs, leading to a steep initial decrease in Ag content in the upper layer of the suspension and a slowdown afterwards (Fig. 5). In Ca(NO_3_)_2_ solutions, complete sedimentation was achieved in half an hour (Fig. 5). As TEM graphs showed, individual AgNPs assembled into compact clusters that sedimented much faster due to their larger sizes (Fig. 4).

Judging from the similar shapes of UV–Vis absorption spectra of supernatant and monodispersed AgNP suspensions, coupled with negligible absorption in the long wavelength region that is characteristic for aggregates (Fig. 6), we can conclude that aggregates but not all individual AgNPs were sedimented by centrifugation. Increasing Ca(NO_3_)_2_ concentrations enhanced the aggregation rate and therefore the sedimentation rate of AgNPs (Fig. 6a). When the Ca(NO_3_)_2_ concentration was larger than the CCC, the fast aggregation regime was obtained and virtually all AgNPs precipitated, making this pre-aggregation approach applicable to a broad range of AgNPs concentrations (Fig. 6b). Furthermore, this approach should be extendable to other AgNPs such as citrate- and PVP-capped AgNPs since they also aggregate in Ca^2+^ and other electrolyte solutions but may require higher concentrations of Ca^2+^ (Huynh and Chen 2011).

### Comparing the aggregation–centrifugation method with ultrafiltration

Both the aggregation–centrifugation method and ultrafiltration enabled measurement of the dissolved Ag concentration in AgNP suspensions. Larger dissolved Ag concentrations were obtained by aggregation–centrifugation than by ultrafiltration (Fig. 7). This suggests that some of the dissolved Ag is not effectively separated from the AgNPs by ultrafiltration. The various Ag species in AgNP suspensions include nanoparticulate Ag, free Ag^+^ in the bulk liquid (Kennedy et al. 2010), Ag bound to ions or organic groups in the bulk liquid (Levard et al. 2013; Liu et al. 2010; Ostermeyer et al. 2013) and Ag^+^ attached to the nanoparticle surface (Pfeiffer et al. 2014). During ultrafiltration, nano-Ag together with any attached Ag^+^ would presumably be retained by the membrane. Silver loss also occurs due to the adsorption of Ag^+^ to the filter units during ultrafiltration. Silver ion adsorption by the same brand of ultrafiltration units has been reported in several studies (Kennedy et al. 2010; Leclerc and Wilkinson 2014; Shen et al. 2015). In the aggregation–centrifugation method, Ag loss by adsorption is minimized. Presumably, all free Ag^+^ in the bulk liquid will remain in the supernatant. Once individual AgNPs aggregate into compact clusters by Ca^2+^ bridging, the dissolved Ag^+^ ions that acted as counter ions to balance the negative charges on AgNP surfaces might become released from the AgNPs. Additionally, Ca^2+^ could replace the surface-attached Ag^+^ in the diffuse and Stern layer, releasing Ag^+^ into the bulk liquid. Therefore, more dissolved Ag can be collected by centrifugation after aggregation. Since both of these pools of Ag^+^ species contribute to the toxicity of AgNPs, it is advantageous to include their concentration in measurements of dissolved Ag in AgNP suspensions.

## Conclusions

In this study, a new method to measure dissolved Ag in AgNP suspensions was developed. By combining aggregation with centrifugation, only half an hour was required to separate dissolved Ag from AgNPs. Uncoated AgNPs were aggregated by Ca(NO_3_)_2_, and a concentration of 2 mM was sufficient to induce the formation of large AgNPs clusters. This pre-aggregation facilitated the sedimentation of a wide range of AgNP concentrations by centrifugation. The combined aggregation–centrifugation method avoided the loss of Ag in ultrafiltration. That more dissolved Ag was obtained by the new method has significant implications for the study of AgNPs toxicity. Since the combined aggregation–centrifugation method significantly reduces centrifugation time to separate nanoparticles from ions, it will be especially helpful for real-time toxicity assays where speed and the convenience of table top centrifugation represent a major methodological improvement. The method may also be applicable for separating ions from other nanoparticles.

## Electronic supplementary material

Below is the link to the electronic supplementary material.

Supplementary material 1 (DOCX 253 kb)

## References

[CR1] Adeleye AS, Conway JR, Garner K, Huang Y, Su Y, Keller AA (2016). Engineered nanomaterials for water treatment and remediation: costs, benefits, and applicability. Chem Eng J.

[CR2] Alexander CM, Dabrowiak JC, Goodisman J (2013). Gravitational sedimentation of gold nanoparticles. J Colloid Interface Sci.

[CR3] Baalousha M, Nur Y, Romer I, Tejamaya M, Lead JR (2013). Effect of monovalent and divalent cations, anions and fulvic acid on aggregation of citrate-coated silver nanoparticles. Sci Total Environ.

[CR4] Bonaccorso F, Zerbetto M, Ferrari AC, Amendola V (2013). Sorting nanoparticles by centrifugal fields in clean media. J Phys Chem C.

[CR5] Chaloupka K, Malam Y, Seifalian AM (2010). Nanosilver as a new generation of nanoproduct in biomedical applications. Trends Biotechnol.

[CR6] Eckhardt S, Brunetto PS, Gagnon J, Priebe M, Giese B, Fromm KM (2013). Nanobio silver: its interactions with peptides and bacteria, and its uses in medicine. Chem Rev.

[CR7] El Badawy AM, Luxton TP, Silva RG, Scheckel KG, Suidan MT, Tolaymat TM (2010). Impact of environmental conditions (pH, ionic strength, and electrolyte type) on the surface charge and aggregation of silver nanoparticles suspensions. Environ Sci Technol.

[CR8] Gorham J, Rohlfing A, Lippa K, MacCuspie R, Hemmati A, David Holbrook R (2014). Storage wars: how citrate-capped silver nanoparticle suspensions are affected by not-so-trivial decisions. J Nanopart Res.

[CR9] Hendel T, Wuithschick M, Kettemann F, Birnbaum A, Rademann K, Polte J (2014). In situ determination of colloidal gold concentrations with UV–Vis spectroscopy: limitations and perspectives. Anal Chem.

[CR10] Huynh KA, Chen KL (2011). Aggregation kinetics of citrate and polyvinylpyrrolidone coated silver nanoparticles in monovalent and divalent electrolyte solutions. Environ Sci Technol.

[CR11] Ivask A (2013). Toxicity mechanisms in *Escherichia coli* vary for silver nanoparticles and differ from ionic silver. ACS Nano.

[CR12] Kennedy AJ (2010). Fractionating nanosilver: importance for determining toxicity to aquatic test organisms. Environ Sci Technol.

[CR13] Kim BYS, Rutka JT, Chan WCW (2010). Current concepts: nanomedicine. N Engl J Med.

[CR14] Leclerc S, Wilkinson KJ (2014). Bioaccumulation of nanosilver by *Chlamydomonas reinhardtii*—nanoparticle or the free ion?. Environ Sci Technol.

[CR15] Levard C, Mitra S, Yang T, Jew AD, Badireddy AR, Lowry GV, Brown GE (2013). Effect of chloride on the dissolution rate of silver nanoparticles and toxicity to *E. coli*. Environ Sci Technol.

[CR16] Li X, Lenhart JJ, Walker HW (2012). Aggregation kinetics and dissolution of coated silver nanoparticles. Langmuir.

[CR17] Liu J, Hurt RH (2010). Ion release kinetics and particle persistence in aqueous nano-silver colloids. Environ Sci Technol.

[CR18] Liu J, Sonshine DA, Shervani S, Hurt RH (2010). Controlled release of biologically active silver from nanosilver surfaces. ACS Nano.

[CR19] Lohse SE, Murphy CJ (2012). Applications of colloidal inorganic nanoparticles: from medicine to energy. J Am Chem Soc.

[CR20] Loza K (2014). The dissolution and biological effects of silver nanoparticles in biological media. J Mater Chem B.

[CR21] McQuillan JS, Shaw AM (2014). Differential gene regulation in the Ag nanoparticle and Ag^+^-induced silver stress response in *Escherichia coli*: a full transcriptomic profile. Nanotoxicology.

[CR22] Meredith HR, Srimani JK, Lee AJ, Lopatkin AJ, You L (2015). Collective antibiotic tolerance: mechanisms, dynamics and intervention. Nat Chem Biol.

[CR23] Michen B, Geers C, Vanhecke D, Endes C, Rothen-Rutishauser B, Balog S, Petri-Fink A (2015) Avoiding drying-artifacts in transmission electron microscopy: Characterizing the size and colloidal state of nanoparticles. Sci Rep 5:9793. http://www.nature.com/articles/srep09793#supplementary-information. doi:10.1038/srep09793 10.1038/srep09793 PMC4428270 25965905

[CR24] Mitrano DM, Barber A, Bednar A, Westerhoff P, Higgins CP, Ranville JF (2012). Silver nanoparticle characterization using single particle ICP-MS (SP-ICP-MS) and asymmetrical flow field flow fractionation ICP-MS (AF4-ICP-MS). J Anal At Spectrom.

[CR25] Morones-Ramirez JR, Winkler JA, Spina CS, Collins JJ (2013). Silver enhances antibiotic activity against gram-negative bacteria. Sci Transl Med.

[CR26] Mostowfi F, Indo K, Mullins OC, McFarlane R (2009). Asphaltene nanoaggregates studied by centrifugation. Energy Fuels.

[CR27] Mulfinger L, Solomon SD, Bahadory M, Jeyarajasingam AV, Rutkowsky SA, Boritz C (2007). Synthesis and study of silver nanoparticles. J Chem Educ.

[CR28] Nel AE (2009). Understanding biophysicochemical interactions at the nano–bio interface. Nat Mater.

[CR29] Oberdörster G, Oberdörster E, Oberdörster J (2005). Nanotoxicology: an emerging discipline evolving from studies of ultrafine particles. Environ Health Perspect.

[CR30] Ostermeyer AK, Mumuper CK, Semprini L, Radniecki T (2013). Influence of bovine serum albumin and alginate on silver nanoparticle dissolution and toxicity to *Nitrosomonas europaea*. Environ Sci Technol.

[CR31] Pace HE, Rogers NJ, Jarolimek C, Coleman VA, Gray EP, Higgins CP, Ranville JF (2012). Single particle inductively coupled plasma-mass spectrometry: a performance evaluation and method comparison in the determination of nanoparticle size. Environ Sci Technol.

[CR32] Peretyazhko TS, Zhang Q, Colvin VL (2014). Size-controlled dissolution of silver nanoparticles at neutral and acidic pH conditions: kinetics and size changes. Environ Sci Technol.

[CR33] Pfeiffer C (2014). Interaction of colloidal nanoparticles with their local environment: the (ionic) nanoenvironment around nanoparticles is different from bulk and determines the physico-chemical properties of the nanoparticles. J R Soc Interface.

[CR34] Piddock LJV (2012). The crisis of no new antibiotics—what is the way forward?. Lancet Infect Dis.

[CR35] Polte J (2012). Formation mechanism of colloidal silver nanoparticles: analogies and differences to the growth of gold nanoparticles. ACS Nano.

[CR36] Schneider CA, Rasband WS, Eliceiri KW (2012). NIH Image to ImageJ: 25 years of image analysis. Nat Methods.

[CR37] Shen M-H, Zhou X-X, Yang X-Y, Chao J-B, Liu R, Liu J-F (2015). Exposure medium: key in identifying free Ag^+^ as the exclusive species of silver nanoparticles with acute toxicity to *Daphnia magna*. Sci Rep.

[CR38] Silvera Batista CA, Larson RG, Kotov NA (2015). Nonadditivity of nanoparticle interactions. Science.

[CR39] Sprenger M, Fukuda K (2016). New mechanisms, new worries. Science.

[CR40] Van Hyning DL, Zukoski CF (1998). Formation mechanisms and aggregation behavior of borohydride reduced silver particles. Langmuir.

[CR41] Van Hyning DL, Klemperer WG, Zukoski CF (2001). Silver nanoparticle formation: predictions and verification of the aggregative growth model. Langmuir.

[CR42] Xiu Z, Zhang Q, Puppala HL, Colvin VL, Alvarez PJJ (2012). Negligible particle-specific antibacterial activity of silver nanoparticles. Nano Lett.

[CR43] Zhang W, Yao Y, Sullivan N, Chen YS (2011). Modeling the primary size effects of citrate-coated silver nanoparticles on their ion release kinetics. Environ Sci Technol.

[CR44] Zhang W, Crittenden J, Li K, Chen Y (2012). Attachment efficiency of nanoparticle aggregation in aqueous dispersions: modeling and experimental validation. Environ Sci Technol.

