# Supplementary material for: New, rapid method to measure dissolved silver concentration in silver nanoparticle suspensions by aggregation combined with centrifugation
Source: J Nanopart Res. 2016 Aug 29;18(9):259. doi: 10.1007/s11051-016-3565-0 (PMC5003901; doi:10.1007/s11051-016-3565-0)
Supplement: Supplementary file 1 — Supplementary material 1 (DOCX 253 kb) [file 11051_2016_3565_MOESM1_ESM.docx]

**Electronic Supplementary Material**

**Journal of Nanoparticle Research**

**New, rapid method to measure dissolved silver concentration in silver nanoparticle suspensions by aggregation combined with centrifugation**

Feng Dong ∙ Eugenia Valsami-Jones ∙ Jan-Ulrich Kreft

Feng Dong (corresponding author)

Institute of Microbiology and Infection & School of Biosciences, University of Birmingham, Edgbaston, Birmingham, B15 2TT, UK

E-mail: [fengdongub@gmail.com](mailto:fengdongub@gmail.com)

Telephone: +44 (0)121 414 8850

Eugenia Valsami-Jones

School of Geography, Earth and Environmental Sciences, University of Birmingham, Edgbaston, Birmingham, B15 2TT, UK

Jan-Ulrich Kreft

Institute of Microbiology and Infection & School of Biosciences, University of Birmingham, Edgbaston, Birmingham, B15 2TT, UK


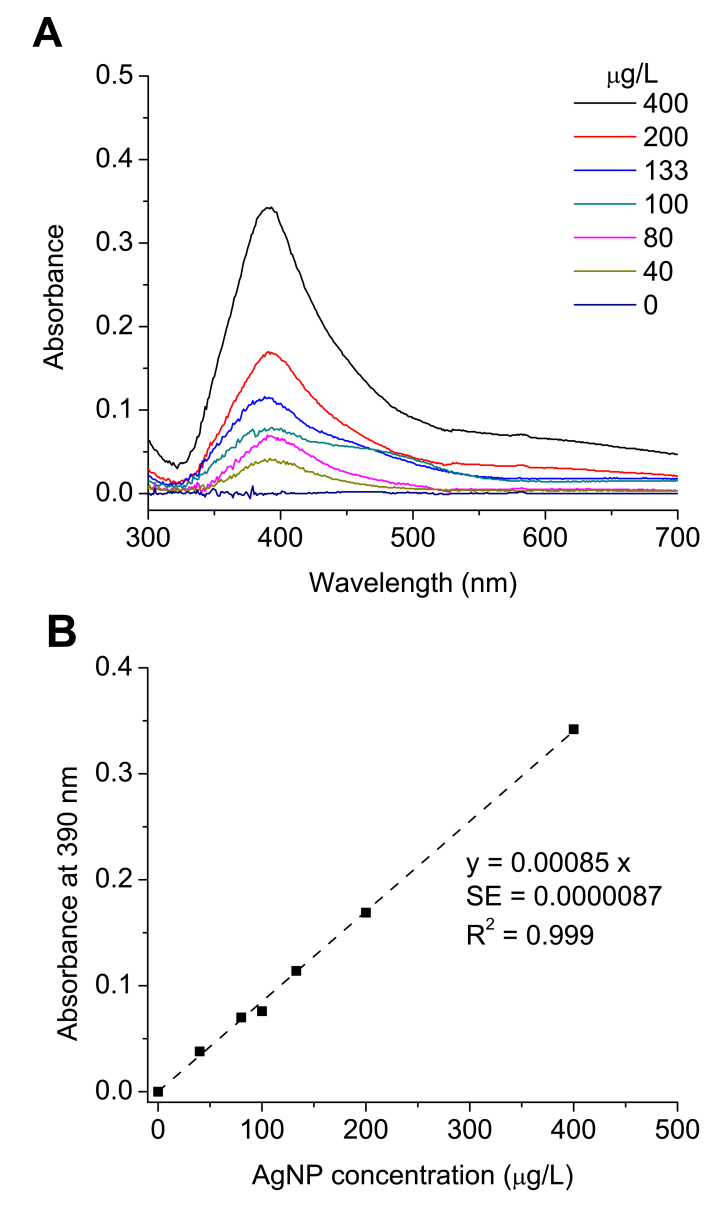


**Fig. S1** **a** UV-Vis absorption spectra of different concentrations of AgNPs. The shapes of the absorption spectra were the same, and all had the peak absorbance at 390 ± 1 nm. **b** Linear regression of the absorbance at 390 nm versus AgNP concentration. SE represents the standard error of the slope.


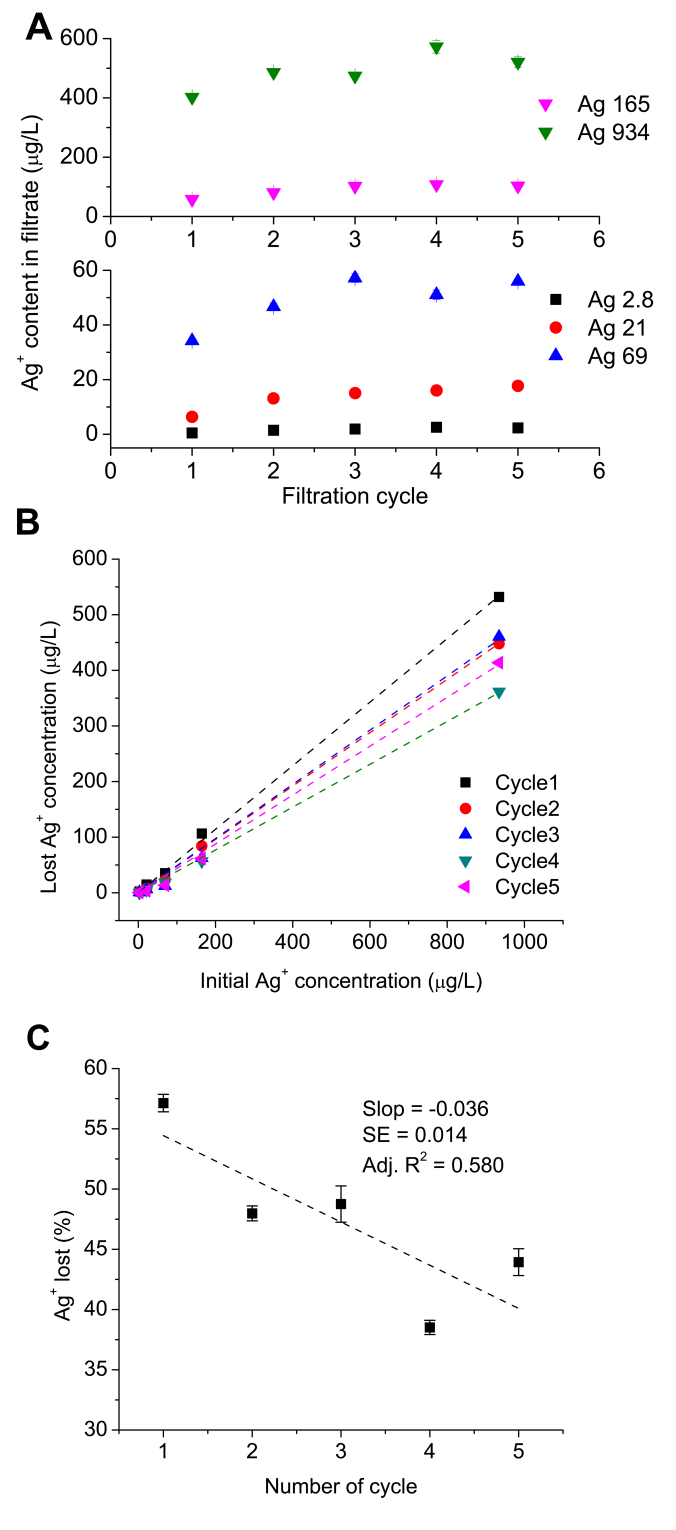


**Fig. S2** Adsorption of Ag^+^ to ultrafiltration units during ultrafiltration. **a** Ag^+^ concentrations in filtrates during five cycles of filtration by centrifugation (4 °C, 4000 g, 20 minutes) with one of five different concentrations of AgNO_3_ (2.8, 21, 69, 165 and 934 µg/L Ag^+^). After each filtration step, filtrates were collected and the Ag^+^ concentration in the filtrates measured by GFAAS. Error bars indicate the standard deviations of three measurements of the sample by GFAAS. **b** Linear regression of Ag^+^ loss versus initial Ag^+^ concentration for each cycle of filtration. The intercept was set to zero. **c** Linear regression of the slopes of the linear regressions in panel b versus filtration cycle. The negative slope of this regression line indicates that the fraction of Ag^+^ lost decreased somewhat with each cycle. The error bars represent standard errors of the slopes.





**Fig. S3** Simulation of sedimentation of AgNPs by centrifugation according to (Bonaccorso et al. 2013) assuming spherical particles. The distance settled after 30 minutes was plotted against the diameter of AgNPs. The parameters for simulation were set as follows: density of AgNPs 10.49 g/cm^3^, density of medium 1 g/cm^3^, liquid viscosity 1 mPa·s, angular velocity of centrifugation 14,000 rpm and rotor radius 9.2 cm.

## References

Bonaccorso F, Zerbetto M, Ferrari AC, Amendola V (2013) Sorting Nanoparticles by Centrifugal Fields in Clean Media. J Phys Chem C 117:13217-13229. doi:10.1021/jp400599g
